# Correction: Radioprotectant Activity of 5-Diethylsulfonamoylsalicylatocopper(II) in Gamma Irradiated Mice

**DOI:** 10.1155/MBD.1999.193

**Published:** 1999

**Authors:** John A. Kuykendall, III, Hugh Simmons, III, Henry J. Irving, Mark A. Wear, Paul G. Sorenson, Linda G. Tipton, Kenchasha M. Maddox, Elsie L. Williams, Van Anh Pham-Tran, Chassie Credit, Israt L. Chowdhury, Shaheen Khan, J. Brooks Tipton, William M. Willingham, John R. J. Sorenson


					CORRECTION
Metal-Based Drugs 6(2) (1999), 127
RADIOPROTECTANT ACTIVITY OF
5-DIETHYLSULFONAMOYLSALICYLATOCOPPER(II)
IN GAMMA IRRADIATED MICE
John A. Kuykendall, III, Hugh Simmons, III, Henry J. Irving, Mark A. Wear,
Paul G. Sorenson, Linda G. Tipton, Kenchasha M. Maddox, Elsie L. Williams,
Van Anh Pham-Tran, Chassie Credit, Israt L. Chowdhury, Shaheen Khan,
J. Brooks Tipton, William M. Willingham, and John R.J. Sorenson*
Figures 2 and 3 were permuted. The right figures with their appropriate legends are
pasted below:
lOO
90
r"
8o
7o
-.I 60
50
30
5 10 15 20 25
TIME AFTER IRRADIATION(DAY)
30
Figure 2. Survival of 8.0 Gy whole-body irradiated mice treated with vehicle (O), 40 tmol (), 60 larnol
(B), or 80 lamol (a,) Cu(II)(5-DESS)/kg of body mass.
100
70
40
5 10 15 20 25
TIME AFTER IRRADIATION(DAY)
Figure 3. Survival of 8.0 Gy whole-body irradiated mice treated with vehicle (O), $0 tmol(), 100 Ixmol
(B), or 120 Ixmol (a,) Cu(II)(5-DESS)/kg ofbody mass.
193

				

